# Mendelian randomization analyses explore the effects of micronutrients on different kidney diseases

**DOI:** 10.3389/fnut.2024.1440800

**Published:** 2024-09-13

**Authors:** Chengdong Shi, Hongliang Cao, Guoqiang Zeng, Hao Wu, Yuantao Wang

**Affiliations:** Department of Urology II, The First Hospital of Jilin University, Changchun, China

**Keywords:** kidney diseases, micronutrients, Mendelian randomization, causal effect, genome-wide association studies

## Abstract

**Background:**

The impact of micronutrients, including vitamins and minerals, on different kidney diseases has been reported in some observational studies; however, their causal relationship remains uncertain. We aimed to ascertain the causal genetic relationships between micronutrients and different kidney diseases using the Mendelian randomization (MR) method.

**Methods:**

Instrumental variables (IVs) for genetically predicting calcium (Ca), iron (Ir), Zinc (Zn), selenium (Se), copper (Cu), vitamin D (Vit D), and vitamin C (Vit C) levels in humans were obtained, and a bidirectional two-sample MR was used to examine potential associations between the levels of these seven micronutrients and the risk of seven different kidney diseases including hypertensive renal disease, diabetic nephropathy, IgA nephropathy, membranous nephropathy, cystic nephropathy, chronic kidney disease (CKD), and chronic tubulo-interstitial nephritis. Five different MR analyses were conducted, with the main method being the inverse variance-weighted (IVW) method. Moreover, sensitivity analyses were performed to assess heterogeneity and potential pleiotropy.

**Results:**

The IVW method revealed that Ca levels were associated with a decreased risk of hypertensive renal disease (OR = 0.61, 95% CI: 0.40–0.93, *p*-value = 0.022), and Se levels were associated with a decreased risk of hypertensive renal disease (OR = 0.72, 95% CI: 0.53–0.99, *p*-value = 0.040), diabetic nephropathy (OR = 0.83, 95% CI: 0.73–0.93, *p*-value = 0.002), and CKD (OR = 0.87, 95% CI: 0.77–0.99, *p*-value = 0.028). Conversely, Vit D levels were associated with an increased risk of polycystic kidney disease (OR = 1.76, 95% CI: 1.15–2.69, *p*-value = 0.0095). In addition, no potential causal relationship was found between vitamin C levels, iron levels, zinc levels, and copper levels and different kidney diseases. Meanwhile, inverse Mendelian randomization showed no potential causal relationship between different chronic kidney diseases and micronutrients. The Cochrane’s Q test, MR-Egger regression, and MR-PRESSO did not suggest heterogeneity and pleiotropy, providing evidence of the validity of the MR estimates.

**Conclusion:**

Our results indicate a cause-and-effect connection between micronutrients and certain kidney diseases, but additional study is required to provide more conclusive evidence. This research has the potential to assist clinicians in managing the consumption of specific micronutrients among individuals with chronic kidney diseases, as well as in promoting disease prevention among both healthy populations and those who are susceptible to chronic underlying conditions.

## Introduction

Kidney diseases are a general term that encompasses a variety of disease types with numerous classification criteria, including primary glomerular diseases (such as membranous nephropathy, IgA nephropathy, glomerulonephritis), acute kidney failure, chronic tubulointerstitial nephritis, diabetic nephropathy, hypertensive renal disease, polycystic kidney disease, and chronic kidney disease (CKD), among others (1). According to the International Society of Nephrology, at least 850 million people worldwide suffer from kidney diseases. In addition, The global prevalence of CKD varies between 8 and 16%, as reported by the International Society of Nephrology. In 2010, CKD ranked 18th among all causes of death worldwide, with an annual mortality rate of 16.3 deaths per 100,000 individuals. This rate is rising, presenting a significant challenge to the global public health system (2, 3). The pathogenic mechanisms of different kidney diseases are complex and not entirely understood due to their numerous origins and consequences. In the absence of appropriate intervention and therapy, the condition has the potential to rapidly advance towards end-stage renal failure.

Micronutrients, which encompass minerals and vitamins, typically comprise non-metallic or metallic components, including calcium (Ca), iron (Ir), Zinc (Zn), selenium (Se), copper (Cu), as well as both fat-soluble and water-soluble vitamins. These nutrients are necessary for normal growth, development, and physiological activities, even if they are required in smaller amounts (4). However, the available data indicates that almost 30% of the global population experiences at least one kind of trace nutrient deficiency, which primarily affects children and pregnant women (5). Furthermore, observational studies have revealed the correlation between micronutrients and different kidney diseases. A prospective study conducted by Chen CY et al. found a positive correlation between serum Se and Zn levels and renal function, as well as improved kidney outcomes. Specifically, higher Zn concentrations were found to independently predict better kidney survival (6). In addition, cohort research conducted over a period of 12 years demonstrated correlations between inadequate consumption of phosphorus, vitamin B2, and folate in one’s diet and elevated consumption of vitamin B6 and vitamin C in one’s diet, with an elevated susceptibility to CKD stage 3B and higher (7). Additionally, vitamin D and its metabolites play an important role in regulating calcium-phosphorus balance as well as bone formation and calcification. They are associated with the risk of various diseases and complications. A meta-analysis of observational studies showed that CKD patients treated with vitamin D had a lower mortality rate compared to those who did not receive treatment (8).

Mendelian randomization (MR) analysis is an effective epidemiological method that uses genetic variation as instrumental variables (IVs) to analyze causal relationships between exposure factors and outcomes (9). Mendel’s law of inheritance posits that genetic differences are predetermined and independent of postnatal conditions, significantly mitigating the impact of common confounding variables and the potential for reverse causation (10, 11). In this study, MR analyses were conducted to investigate the causal relationships between trace nutrients and different kidney diseases.

## Materials and methods

### Study design

**Figure 1:**
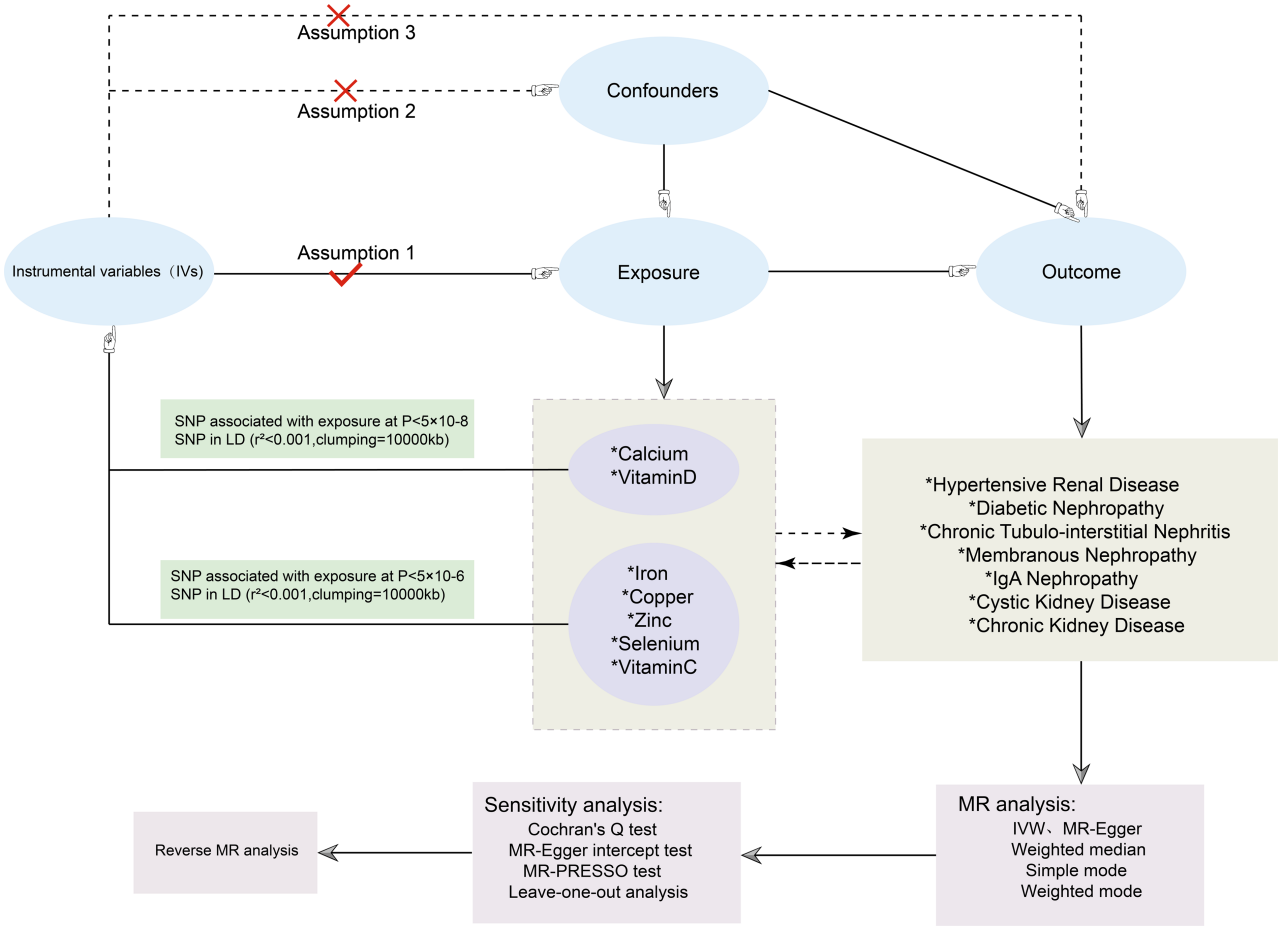
Flowchart of the MR investigating the causal relationship between serum micronutrients and different kidney diseases. SNPs, Single nucleotide polymorphisms; LD, linkage disequilibrium; MR, Mendelian randomization.

The STROBE-MR (Strengthening Reporting of Observational Studies in Epidemiology Using Mendelian Randomization) statement was followed in the conduct of our study (12). In this study, serum levels of Cu, Ir, Zn, Ca, Se, Vitamin C (Vit C), and Vitamin D (Vit D) were considered as exposures to seven different kidney diseases, including hypertensive renal disease, diabetic kidney disease, IgA nephropathy, membranous nephropathy, polycystic kidney disease, different kidney disease (CKD), and chronic tubulointerstitial nephritis as outcomes. The screening of MR is contingent upon the rigorous adherence to three fundamental assumptions (1): a robust correlation exists between the chosen IVs and the factors of exposure (2); The IVs do not exhibit any associations with confounding factors that could potentially influence the outcome (3); The IVs can solely impact outcomes via the exposure factor route, and there is no direct association between IVs and outcomes (13). The data analysis in this study relied solely on publicly accessible data, obviating the need for ethical approval from an ethics commission (Figure 1).

### GWAS statistics source

The micronutrient data utilized in this work were obtained from the IEU OpenGWAS program and are accessible at the following website: https://gwas.mrcieu.ac.uk/. The FinnGen Consortium (website: https://www.finngen.fi/en) provides Genome-wide association studies (GWAS) for seven different kidney diseases. The study included exclusively European populations as participants, and there was no overlap in the samples between exposures and outcomes. This approach significantly minimized mistakes caused by confounding factors. In cases where multiple independent GWAS were accessible, the study with a greater number of participants was chosen. Pooled GWAS data were chosen for the analysis of five important minerals and two vitamins (14, 15). The detailed information of GWAS on exposures and outcomes used in this MR is shown in Table 1.

**Table 1:** The detailed information of GWAS on exposures and outcomes incorporated in this MR.

GWAS ID	Population	Trait	Consortium	Sample size	Number of SNPs
ebi-a-GCST90025990	European	Calcium levels	NA	400,792	4,218,949
ebi-a-GCST90025967	European	Vitamin D levels	NA	418,691	4,225,238
ieu-a-1073	European	Copper	NA	2,603	2,543,646
ieu-a-1049	European	Iron	GIS	23,986	2,096,457
ieu-a-1077	European	Selenium	NA	2,603	2,543,617
ieu-a-1079	European	Zinc	NA	2,603	2,543,610
met-a-348	European	Ascorbate (Vitamin C)	NA	2,085	2,545,101
finn-b-I9_HYPTENSRD	European	Hypertensive renal disease	FinnGen_r5	163,305	16,380,163
finn-b-DM_NEPHROPATHY_EXMORE	European	Diabetic nephropathy	FinnGen_r5	184,987	16,380,336
finn-b-N14_CHRONTUBULOINTNEPHRITIS	European	Chronic tubulointerstitial nephritis	FinnGen_r5	201,648	16,380,412
ebi-a-GCST010005	European	Membranous nephropathy	NA	7,979	5,327,688
ebi-a-GCST90018866	European	IgA nephropathy	NA	477,784	24,182,646
finn-b-Q17_CYSTIC_KIDNEY_DISEA	European	Cystic kidney disease	FinnGen_r5	218,448	16,380,465
finn-b-N14_CHRONKIDNEYDIS	European	Chronic kidney disease	FinnGen_r5	216,743	16,380,459

### Selection of the IVs

Typically, we set the threshold at *p* < 5 × 10^−8^ in order to identify single Nucleotide Polymorphisms (SNPs) that are substantially associated with exposures. Nevertheless, certain summarized GWAS datasets exhibited a restricted number of SNPs that achieved genome-wide significance. Consequently, we choose to lower the criterion for certain datasets. The association thresholds for Ca and Vit D were established at *p* < 5 × 10^−8^, whereas for Cu, Ir, Zn, Se, and VitC, the association thresholds were set at *p* < 5 × 10^−6^. The implementation of various thresholds was employed in earlier MR research with the objective of achieving a higher number of compatible SNPs (16, 17). In order to minimize bias, we then eliminated linkage disequilibrium (LD) and established a threshold of r2 < 0.001, with kb = 10,000. In addition, in order to minimize bias, we eliminated palindromic variants of incompatible alleles and evaluated the potency of the chosen SNPs by computing the F statistic with the following formula:


$$F=R^{2}\times \left(N-2\right)/\left(1-R2\right)$$

where N denotes the sample size, and R2 indicates the extent to which IVs explain the exposure. And we would eliminate the SNPs with *F* < 10 (18, 19). Then, we conducted the Steiger test before each MR analysis to avoid reverse causality and included SNPs with TRUE results. Furthermore, we manually screened these SNPs using PhenoScanner^1^ and excluded any SNPs associated with confounding factors, such as smoking and alcohol, which could potentially affect the study results. Additionally, this study conducted a reverse Mendelian randomization analysis to reduce the potential impact of chronic kidney diseases on micronutrient levels. Since a threshold of *p* < 5 × 10^−8^ did not yield enough SNPs, we also set a threshold of *p* < 5 × 10^−6^ for selecting instrumental variables and proceeded with subsequent analyses.

### MR analyses and sensitivity analyses

**Figure 2:**
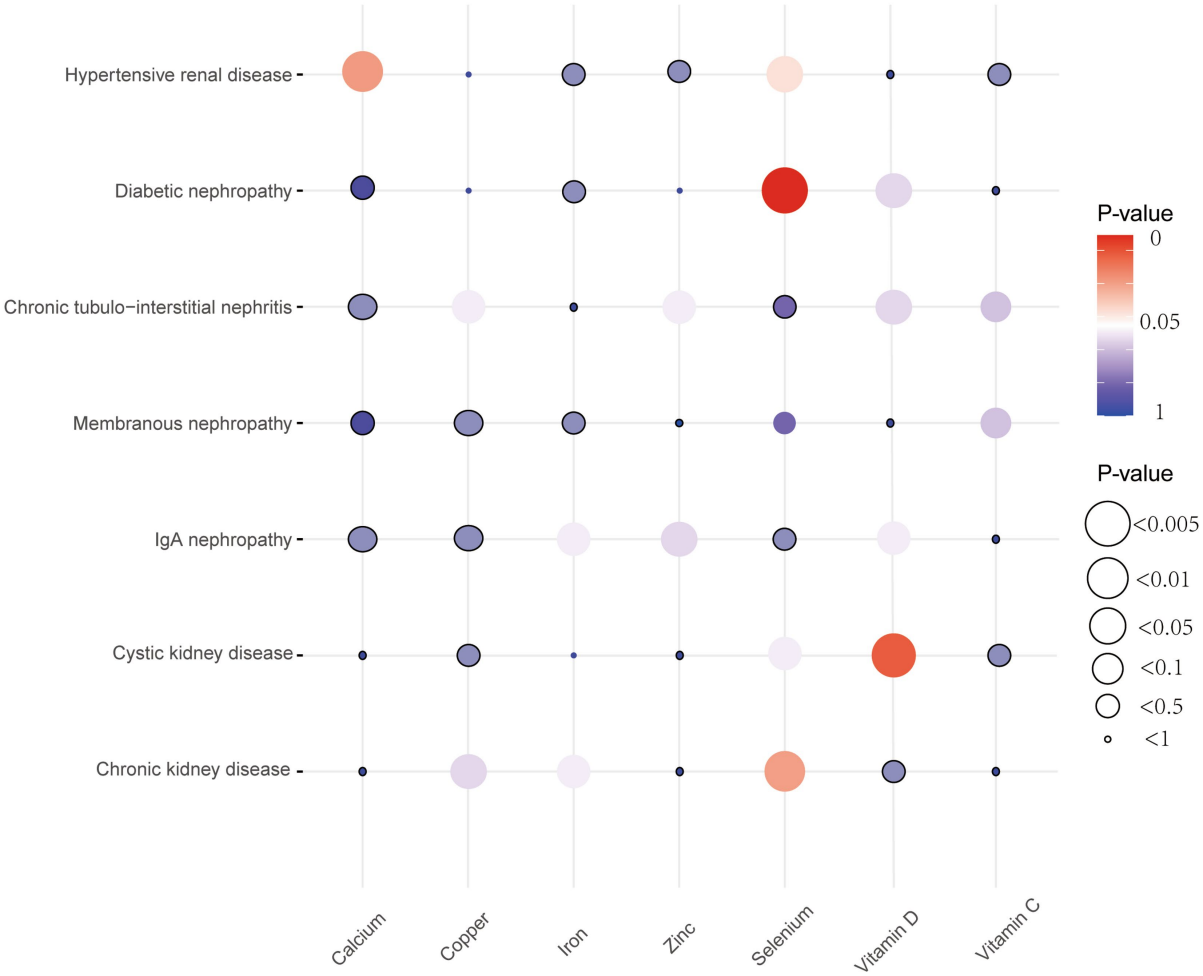
Bubble plots of MR results between micronutrients and seven chronic kidney diseases derived using the IVW method. MR, Mendelian randomization; IVW, inverse variance weighted.

Five different methods of MR, including MR Egger, weighted median, inverse variance weighted (IVW), simple mode, and weighted mode, were utilized to investigate the causal effects between micronutrients and chronic kidney diseases. The basic statistical model employed in this study was IVW estimation, while the remaining four methods were utilized to enhance their robustness. A significance level of less than 0.05 was deemed to be statistically significant. The Cochran’s Q test was employed to evaluate the heterogeneity of results, and a *p*-value of less than 0.05 was utilized to indicate the presence of heterogeneity (20). The assessment and correction of horizontal pleiotropy were conducted using MR-Egger regression and MR-PRESSO, and *p*-value of less than 0.05 was obtained, confirming the presence of horizontal pleiotropy (21). Furthermore, a leave-one-out analysis was conducted to evaluate the potential impact of aberrant SNPs on the obtained results. Funnel plots were employed to evaluate potential directional pleiotropy. The statistical analyses were conducted using R software version 4.3.2, specifically utilizing the TwoSampleMR R package (v0.5.7) and MR-PRESSO (v1.0; Figure 2).

## Results

**Figure 3:**
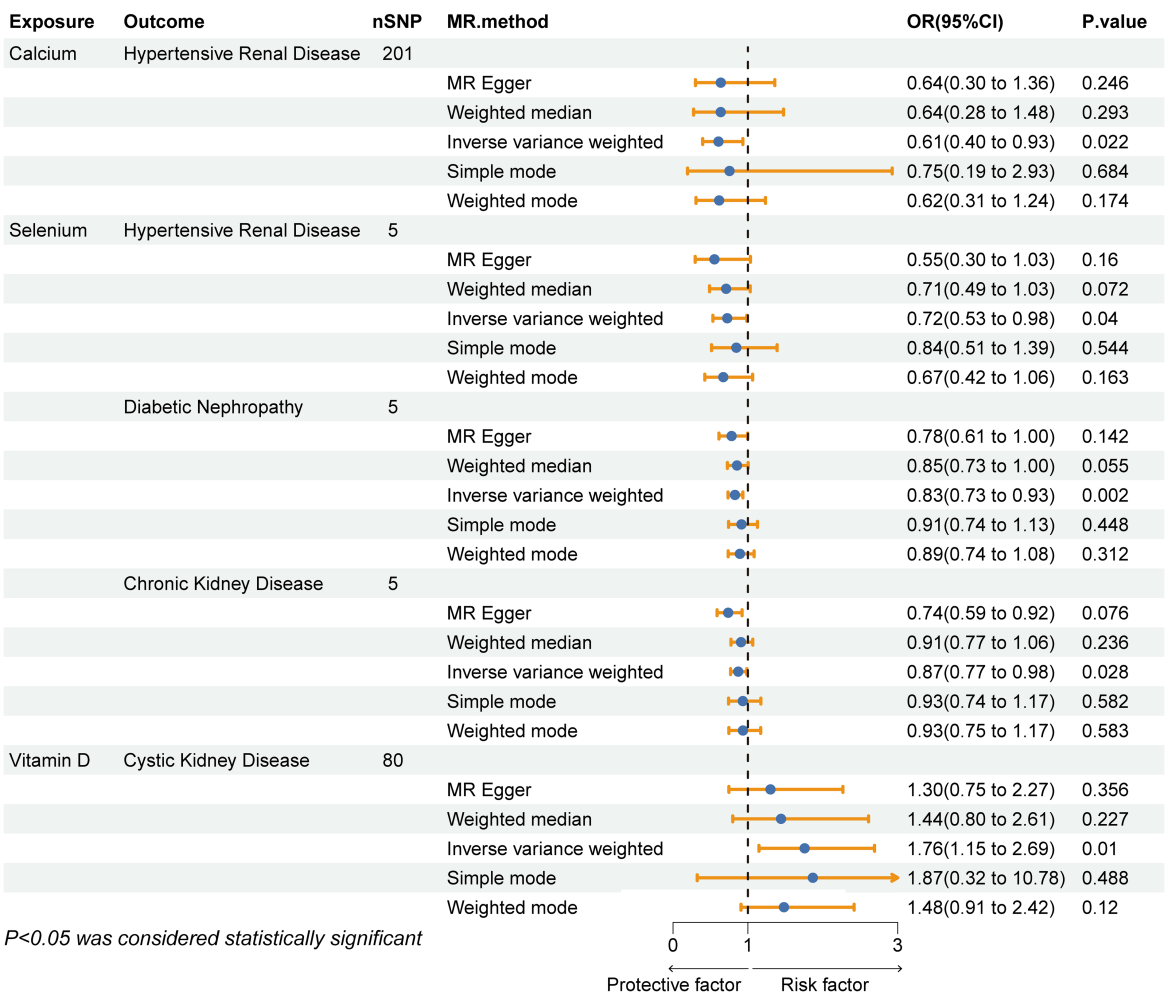
Forest plot showing the causal relationship between micronutrients and chronic kidney disease with a P- value <0.05 calculated using the IVW method. IVW, inverse variance weighting method; OR, odds ratio; CI, confidence interval.

**Figure 4:**
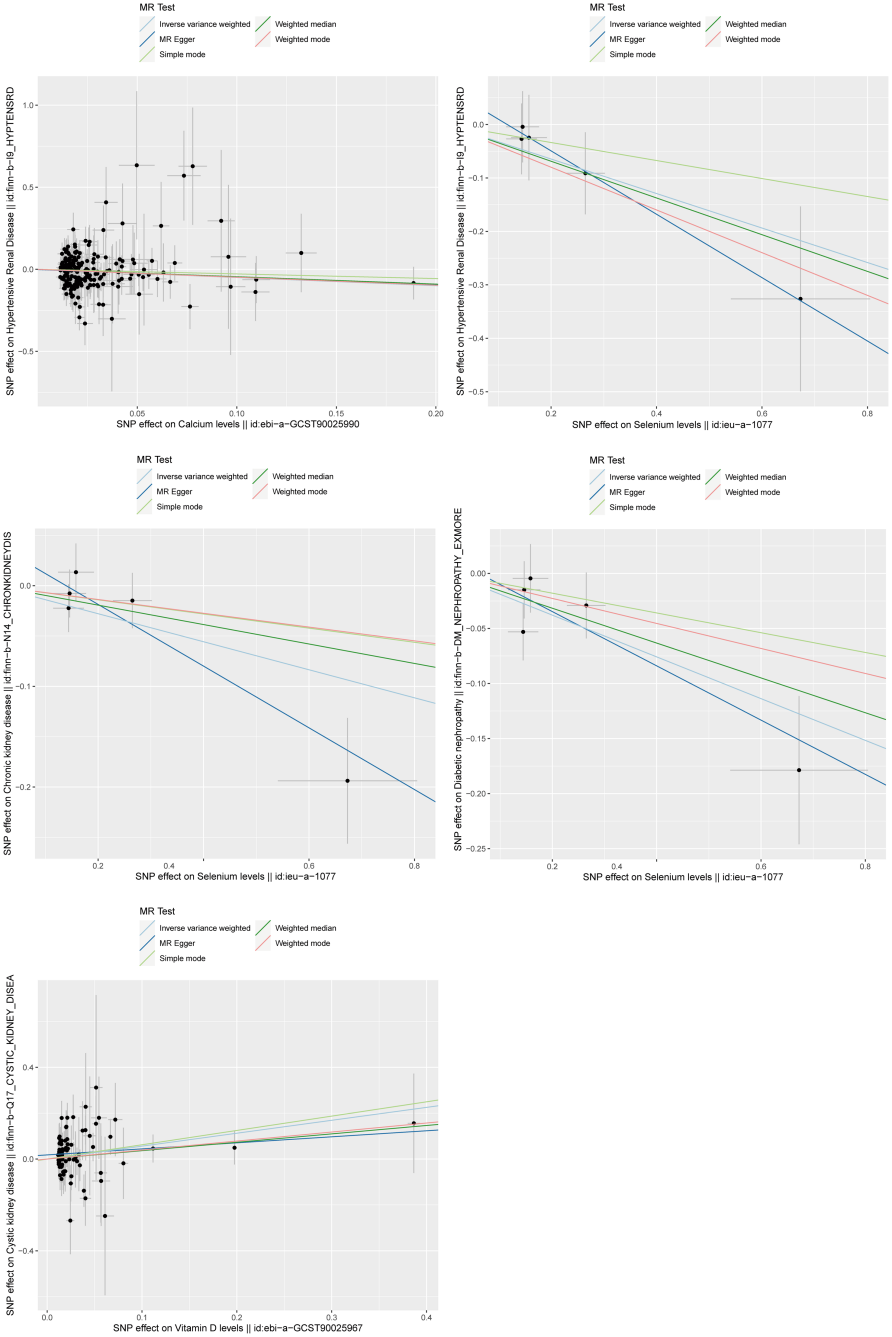
Scatterplot of positive results from MR analysis between exposure and outcome using IVW method. IVW, inverse variance weighted; MR, Mendelian randomization.

After strictly following the IVs screening process, we finally obtained SNPs for this MR Study (Supplementary Table 1). Figures 3, 4 show the positive results of this two-sample MR analysis of micronutrients and seven chronic kidney diseases using the IVW approach. The IVW analysis revealed that Ca was associated with a decreased risk of hypertensive renal disease (OR = 0.61, 95% CI: 0.40–0.93, *p*-value = 0.022), and Se was associated with a decreased risk of hypertensive kidney disease (OR = 0.72, 95% CI: 0.53–0.99, *p*-value = 0.040), diabetic nephropathy (OR = 0.83, 95% CI: 0.73–0.93, *p*-value = 0.002), and chronic kidney disease (OR = 0.87, 95% CI: 0.77–0.99, *p*-value = 0.028). Conversely, vitamin D levels were associated with an increased risk of polycystic kidney disease (OR = 1.76, 95% CI: 1.15–2.69, *p*-value = 0.0095). In addition, no potential causal relationship was found between vitamin C levels, iron levels, zinc levels, and copper levels and different kidney diseases (Supplementary Tables 4–7). Meanwhile, the results of reverse Mendelian randomization showed no potential causal association between different chronic kidney diseases and micronutrients (*p* > 0.05; Supplementary Table 9). The scatterplot in Figure 4 illustrates the positive outcomes obtained through the IVW method, with a *p*-value < 0.05. The results of the MR analysis for the other exposures and outcomes are presented in Supplementary Tables 2–8. The Cochran’s Q test, which incorporates the IVW and MR-Egger methods, did not indicate any significant heterogeneity, as shown by a *p*-value > 0.05. Additionally, the MR-PRESSO and MR-Egger regressions did not reveal any horizontal pleiotropy with *p*-value > 0.05 (Table 2). Furthermore, the Leave-One-Out analysis yielded no SNPs exhibiting significant bias effects, and the funnel plots demonstrated minimal directional pleiotropy, as depicted in Supplementary Figures 1–5. All these sensitivity analysis results provided evidence of the validity of the MR estimates.

**Table 2:** Sensitivity analyses of MR with positive results by using IVW method.

Micronutrients	Outcome	Sensitivity Analysis	Method	P
Calcium	Hypertensive renal disease	Heterogeneity	Cochran’s Q test MR-Egger	0.393
			Cochran’s Q test (IVW)	0.411
		Pleiotropy	Intercept of MR-Egger test	0.875
			MR- PRESSO global test	0.433
Selenium	Hypertensive renal disease	Heterogeneity	Cochran’s Q test MR-Egger	0.995
			Cochran’s Q test (IVW)	0.908
		Pleiotropy	Intercept of MR-Egger test	0.403
			MR- PRESSO global test	0.903
Selenium	Chronic kidney disease	Heterogeneity	Cochran’s Q test MR-Egger	0.520
			Cochran’s Q test (IVW)	0.282
		Pleiotropy	Intercept of MR-Egger test	0.193
			MR- PRESSO global test	0.294
Selenium	Diabetic nephropathy	Heterogeneity	Cochran’s Q test MR-Egger	0.440
			Cochran’s Q test (IVW)	0.562
		Pleiotropy	Intercept of MR-Egger test	0.636
			MR- PRESSO global test	0.605
Vitamin D	Cystic kidney disease	Heterogeneity	Cochran’s Q test MR-Egger	0.758
			Cochran’s Q test (IVW)	0.708
		Pleiotropy	Intercept of MR-Egger test	0.103
			MR- PRESSO global test	0.700

## Discussion

This study employed MR to examine the potential causal associations between serum levels of micronutrients and the occurrence of different kidney diseases. The study revealed that increased Vit D levels were associated with an elevated risk of cystic nephropathy. Additionally, elevated levels of calcium and selenium were identified as protective factors against Hypertensive renal disease, while selenium levels were found to decrease the risk of diabetic nephropathy and CKD. Nevertheless, our study is subject to several limitations. Firstly, the levels of Ir, Cu, Zn, Se, and Vit C did not produce a sufficient number of single nucleotide polymorphisms (SNPs) for the analysis of MR at a significance level of 5 × 10^−8^. As a result, we changed the threshold criterion to 5 × 10^−6^. However, this adjustment would have reduced the overall significance of our study. It is worth noting that this approach has been commonly used in numerous studies and does not inherently impact the outcomes. Secondly, it is important to note that our study was limited to participants of European descent, potentially impacting the applicability of the findings to other populations. Thirdly, the levels of micronutrients may exert varying degrees of influence on different kidney diseases patients of different genders or ages. However, due to the lack of individual-level information in GWAS data, stratified studies cannot be conducted. Finally, in addition to exploring causal relationships, the quantitative relationship between exposure and outcome is also crucial, as it can provide better clinical guidance. Nevertheless, due to the lack of quantitative data on micronutrients and kidney diseases in the GWAS Catalog, this study cannot further investigate the dose–response relationship between exposure and outcome at this time. It is hoped that specific datasets in this area will be available for further study in the future.

### Potential causal relationship between Ca and hypertensive renal disease

Hypertensive renal disease (Hypertensive Nephropathy, HTN) is a chronic disease characterized by elevated blood pressure and chronic kidney disease, including the presence of protein in the urine and reduced glomerular filtration rate. HTN is believed to arise from prolonged and unregulated hypertension, and it ranks as the second most prevalent factor contributing to the advancement of end-stage renal disease following diabetic nephropathy (22). The primary pathogenic processes of Hypertensive nephropathy involve changes in renal hemodynamics and remodeling of the renal vasculature. Furthermore, previous studies have demonstrated the significance of podocyte damage, epithelial-mesenchymal transition (EMT), and tubulointerstitial fibrosis as pathogenic mechanisms in HTN (23, 24). The present amount of research on the association between calcium levels and hypertensive nephropathy is currently minimal. A meta-analysis of cohort studies revealed a significant association between calcium consumption and a decreased likelihood of HTN. Specifically, for every incremental increase of 500 mg/day in dietary calcium intake, there was an approximate 7% reduction in the risk of HTN (25). Several studies have indicated that a deficiency in calcium levels can induce the secretion of parathyroid hormone (PTH). The type-1 parathyroid hormone receptor (PTHR1)/ Galpha(s)/3′,5′-cyclic adenosine monophosphate (cAMP)/protein kinase A (PKA) pathway is activated by parathyroid hormone to raise the concentration of calcium in vascular smooth muscle cells. This, in turn, leads to an increase in vascular reactivity and an elevation in blood pressure. Furthermore, parathyroid hormone (PTH) stimulates the production of cyclic adenosine monophosphate (cAMP) and the release of renin by PTHR-1, thereby facilitating the synthesis of angiotensin II (Ang II) and aldosterone (26, 27). Increased synthesis of Ang II and aldosterone affects blood pressure regulation. Prolonged elevation of these hormones leads to changes in renal hemodynamics, causing renal arteriole narrowing, increased vascular resistance, and reduced blood circulation. This, in turn, induces vascular remodeling in the kidneys, manifested as thickening of the renal arteriole intima, reduced local renal blood flow, and compensatory hypertrophy of adjacent renal units. Meanwhile, Ang II causes podocyte damage and, as a potent vasoconstrictor in the RAS, it plays a crucial role by inducing various cytokines involved in inflammation, cell proliferation, and tubulointerstitial fibrosis (23, 28). In summary, the sequence of events described leads to the development of microalbuminuria, a reduction in glomerular filtration rate, and ultimately promotes the development of HTN.

### Relationship between Se and hypertensive renal disease and chronic kidney disease

Se is a non-metal element that plays a crucial role in the human body due to its antioxidant properties. It and its amino acid derivatives are involved in various physiological and pathological processes, including oxidative stress and immune-inflammatory responses. The role of selenium in certain kidney diseases has been mentioned in several observational studies (29). A research examining the correlation between selenium consumption and CKD among middle-aged and elderly individuals in China, utilizing data from the Chinese National Health Service (CHNS), indicates that sufficient se intake could potentially exert a beneficial influence on CKD (30). Lifu Lei et al. constructed a rat model with a lack of Se and observed that Se deficiency leads to an increase in the production of hydrogen peroxide by reducing the expression of renal glutathione peroxidase 1. Additionally, it enhances the activity of nuclear factor kappa-B(NF-κB), which in turn increases the expression of renal Angiotensin II type-1 receptor (AT1R), resulting in sodium retention and elevated blood pressure (31). In addition, a study conducted by Liu et al. has revealed that a deficiency in selenium may contribute to the inflammation of renal fibrosis. This deficiency has been observed to facilitate the process of epithelial-mesenchymal transition (EMT) in renal fibrosis, hence promoting the deposition of extracellular matrix (ECM) in this particular condition. The mechanism may involve selenium deficiency activating the PI3K/Akt signaling pathway, leading to the promotion of fibroblast proliferation and collagen synthesis. This activation subsequently regulates the proliferation and survival of epithelial cells, impacts protein synthesis and the cell cycle in renal interstitial cells, and ultimately contributes to the development and advancement of renal fibrosis. In summary, Se plays a crucial role in oxidative stress, EMT, and the renal fibrosis process within the body. It is noteworthy that renal oxidative stress contributes to the development and progression of CKD, and EMT and renal fibrosis are significant pathological processes in the progression of HTN (32, 33). Therefore, consistent with the findings of most observational studies, Se levels are associated with a reduced risk of HTN and CKD.

### Effect of Se levels on diabetic nephropathy

Diabetic kidney disease (DKD) is the primary cause of end-stage renal disease (ESRD), with about 40% of diabetic patients having varying degrees of impaired renal function, and DKD is mainly due to microvascular damage caused by prolonged uncontrolled glucose (34). DKD is characterized by several key pathological symptoms, including the thickening of the basement membranes of the glomeruli and tubules, the loss of podocytes, the dilation of the thylakoid membranes, and the localized degeneration of thylakoid cells and thylakoid stroma, among others (35). Furthermore, the pathophysiological processes responsible for the development of DKD predominantly encompass the production of reactive oxygen species (ROS), nutritional detection, recruitment of inflammatory cells, mitochondrial impairment, tubulointerstitial fibrosis, and various other mechanisms (34, 36, 37). Meanwhile, certain metabolic factors (oxidative stress) and hemodynamic factors (activation of the RAS system) may enhance the effects of common pathogenic mechanisms in the context of hyperglycemia, contributing to the development of DKD (38). Studies have demonstrated that selenium can decrease elevated levels of glucose in the blood and enhance the ability of rats to tolerate glucose. An RCT study conducted on humans demonstrated that administering Se supplements for a duration of 12 weeks had a positive impact on serum insulin levels in patients with DKD (39). A study conducted on rats revealed that a lack of selenium caused oxidative stress through the activation of TGF-β1 in both normal and diabetic rats. In both normal and diabetic rats, the presence of selenium deficiency resulted in an increase in albuminuria. Additionally, normal rats exhibited higher plasma glucose levels, whereas diabetic rats experienced worsened plasma glucose levels. These findings indicate that a lack of selenium can cause an increase in the expression of TGF-β1, impact the levels and management of glucose in the blood, enhance the body’s reaction to oxidative stress, and result in the presence of protein in the urine. Ultimately, these factors contribute to the onset and progression of DKD (40). Although the existing body of information on the association between selenium and kidney illness primarily relies on animal models, it offers valuable insights into the underlying mechanisms in humans. However, additional study and exploration are necessary to understand this relationship fully.

### Association between vitamin D and cystic kidney disease

Cystic kidney disease is categorized into congenital and acquired kidney diseases, including autosomal dominant polycystic kidney disease, autosomal recessive polycystic kidney disease, Meckel syndrome, and simple renal cysts. The latter is characterized by the presence of renal cysts of varying degrees (41). Currently, there are very few studies on the relationship between vitamin D levels and cystic kidney disease, and the connection between them remains unclear. Some scholars believe that fibroblast growth factor 23 (FGF23) plays a significant role in the development of cystic kidney disease. This hormone, primarily synthesized by osteoblasts and osteocytes in the bone, is crucial for maintaining normal kidney function. Phosphate, PTH, calcitriol, and calcium are the primary regulatory variables that influence its secretion (42). A study revealed that individuals with vitamin D deficiency rickets exhibited reduced serum FGF23 levels, which subsequently rose following vitamin D therapy (43). The comprehensive review and meta-analysis conducted by Charoenngam et al. yielded findings indicating that the administration of vitamin D3 supplementation resulted in a considerable elevation of intact FGF23 levels in individuals diagnosed with vitamin D deficiency (44). The study conducted by Spichtig et al. provided evidence of the production of FGF23 in the kidneys of rodents with polycystic kidneys. This production resulted in an elevation of FGF23 levels, which subsequently led to a deterioration in kidney function (45, 46). So, FGF23 might be a key point in the relationship between vitamin D levels and cystic kidney disease. However, further research is needed to elucidate the mechanisms of vitamin D’s effects on cystic kidney disease.

## Conclusion

Our research provides initial findings on the genetic link between micronutrients and specific chronic kidney disorders. The results of this study have the potential to assist clinicians in managing the consumption of specific micronutrients among individuals with chronic kidney diseases, as well as in promoting disease prevention among both healthy populations and those who are susceptible to chronic underlying conditions. Nevertheless, further research is required in order to establish more definitive evidence.

## Data Availability

Publicly available datasets were analyzed in this study. This data can be found at: https://www.finngen.fi/en; https://gwas.mrcieu.ac.uk.

## Footnotes

^1^ http://www.phenoscanner.medschl.cam.ac.uk/phenoscanner
