# Supplementary material for: Mendelian randomization analyses explore the effects of micronutrients on different kidney diseases
Source: Front Nutr. 2024 Sep 13;11:1440800. doi: 10.3389/fnut.2024.1440800 (PMC11428537; doi:10.3389/fnut.2024.1440800)
Supplement: Supplementary file 1 [file Image_1.pdf]

A list of Supporting Information

Supplementary figure 1: (a) Leave-one-out between Ca and Hypertensive Nephropathy; (b) Funnel plot between Ca and Hypertensive Nephropathy

Supplementary figure 2: (a) Leave-one-out between Se and Hypertensive Nephropathy; (b) Funnel plot between Se and Hypertensive Nephropathy

Supplementary figure 3: (a) Leave-one-out between Se and Diabetic Nephropathy; (b) Funnel plot between Se and Diabetic Nephropathy

Supplementary figure 4: (a) Leave-one-out between Se and Chronic Kidney Disease; (b) Funnel plot between Se and Chronic Kidney Disease

Supplementary figure 5: (a) Leave-one-out between Vit D and Cystic Kidney Disease; (b) Funnel plot between Vit D and Cystic Kidney Disease

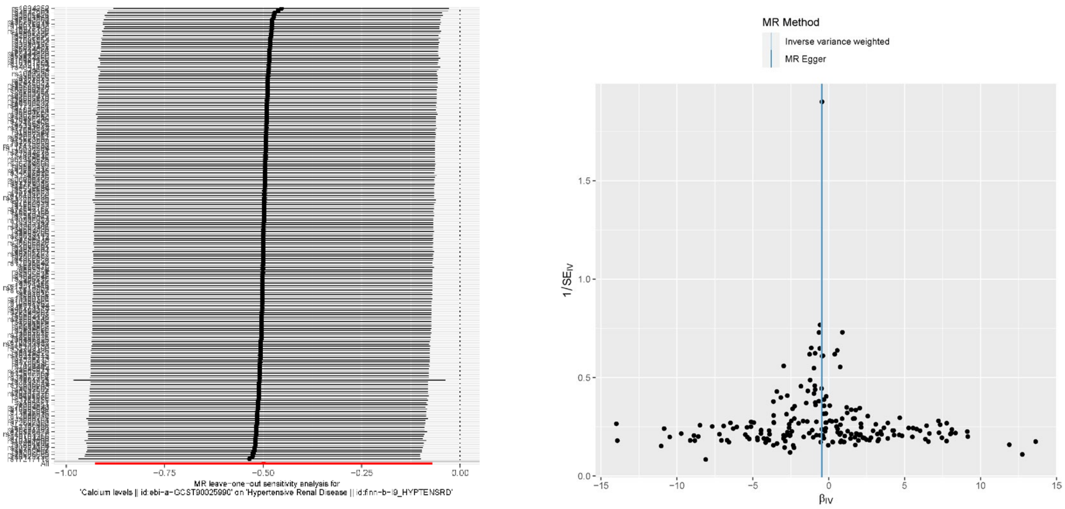

(a) Leave-One-Out plot

(b) Funnel plot

Supplementary figure 1: (a) Leave-one-out between Ca and Hypertensive Nephropathy; (b) Funnel plot between Ca and Hypertensive Nephropathy

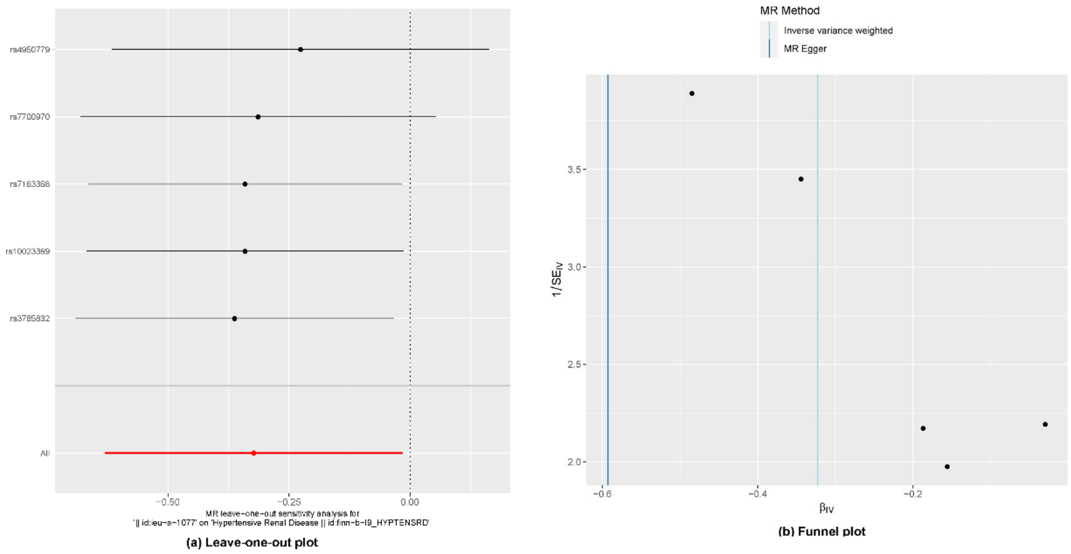

Supplementary figure 2: (a) Leave-one-out between Se and Hypertensive Nephropathy; (b) Funnel plot between Se and Hypertensive Nephropathy

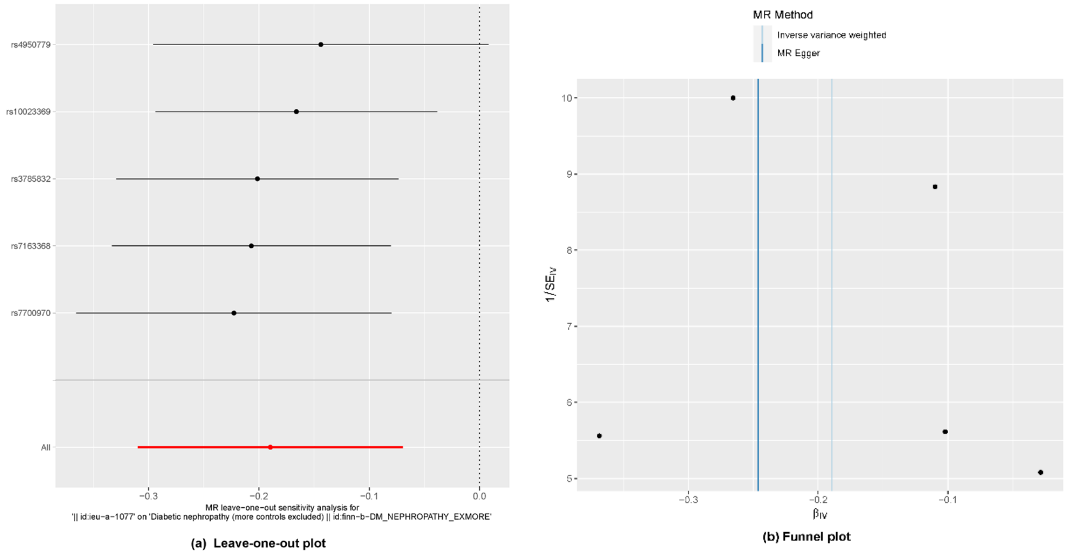

Supplementary figure 3: (a) Leave-one-out between Se and Diabetic Nephropathy; (b) Funnel plot between Se and Diabetic Nephropathy

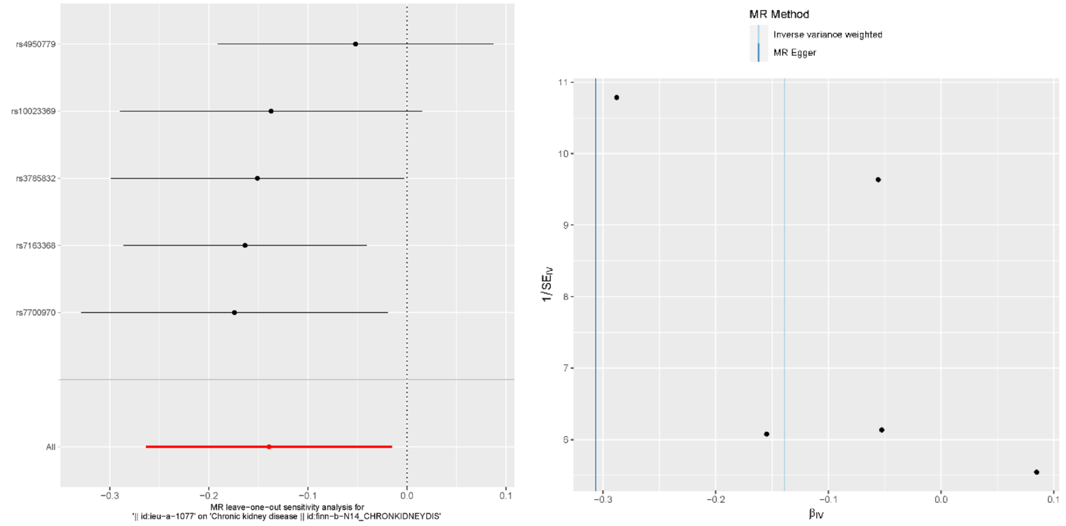

(a) Leave-one-out plot (b) Funnel plot

Supplementary figure 4: (a) Leave-one-out between Se and Chronic Kidney Disease; (b) Funnel plot between Se and Chronic Kidney Disease

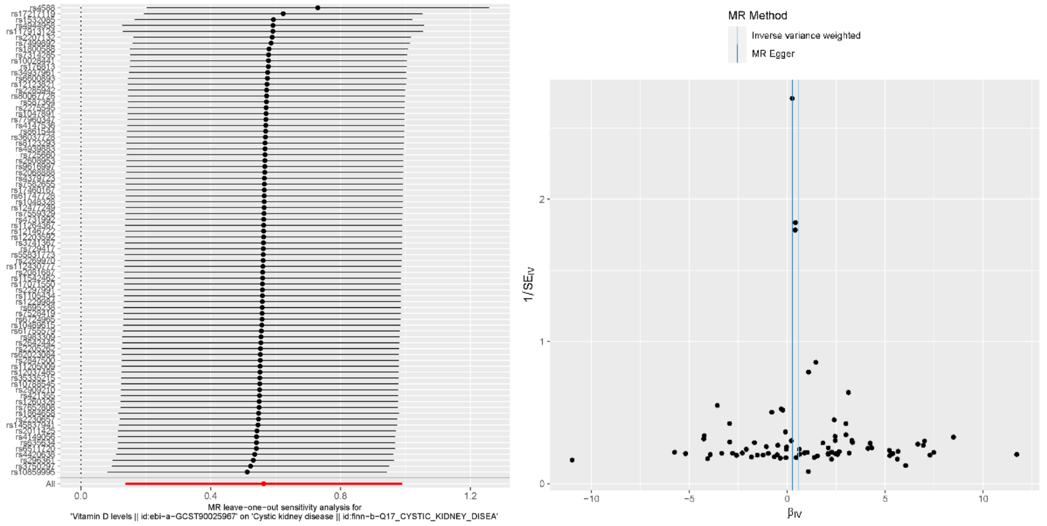

(a) Leave-one-out plot (b) Funnel plot

Supplementary figure 5: (a) Leave-one-out between Vit D and Cystic Kidney Disease; (b) Funnel plot between Vit D and Cystic Kidney Disease
