# Supplementary material for: Mendelian randomization analyses explore the effects of micronutrients on different kidney diseases
Source: Front Nutr. 2024 Sep 13;11:1440800. doi: 10.3389/fnut.2024.1440800 (PMC11428537; doi:10.3389/fnut.2024.1440800)
Supplement: Supplementary file 10 [file Table_9.DOCX]

**Supplementary table 9** Inverse Mendelian randomization results for different chronic kidney diseases and micronutrients.

| Exposure | Outcome | MR method | nSNP | OR (95%CI) | P value |
| --- | --- | --- | --- | --- | --- |
| HTN | Calcium | IVW | 4 | 0.9995(0.9911-1.008) | 0.9099 |
|  |  | MR Egger | 4 | 1.0030(0.9894-1.0169) | 0.7063 |
|  |  | Weighted median | 4 | 1.0016(0.9963-1.0069) | 0.5619 |
|  |  | Simple mode | 4 | 1.0021(0.9962-1.0080) | 0.5412 |
|  |  | Weighted mode | 4 | 1.0017(0.9963-1.0071) | 0.5873 |
|  | Selenium | Wald ratio | 1 | 0.9181(0.7728-1.0907) | 0.3309 |
|  | Iron | Wald ratio | 1 | 1.0160(0.9561-1.0797) | 0.6088 |
|  | Zinc | Wald ratio | 1 | 0.9783(0.8394-1.1401) | 0.7785 |
|  | Copper | Wald ratio | 1 | 1.0024(0.8560-1.1739) | 0.9758 |
|  | Vitamin C | Wald ratio | 1 | 1.0343(0.9686-1.1043) | 0.3138 |
|  | Vitamin D | IVW | 4 | 0.9983(0.9940-1.0027) | 0.4493 |
|  |  | MR Egger | 4 | 1.0007(0.9943-1.0071) | 0.8533 |
|  |  | Weighted median | 4 | 0.9992(0.9937-1.005) | 0.7830 |
|  |  | Simple mode | 4 | 0.9992(0.9918-1.0066) | 0.8434 |
|  |  | Weighted mode | 4 | 0.9994(0.9940-1.0048) | 0.8374 |
| DKD | Calcium | IVW | 4 | 0.9897(0.9759-1.0038) | 0.1507 |
|  |  | MR Egger | 4 | 0.9983(0.9771-1.0200) | 0.8933 |
|  |  | Weighted median | 4 | 0.9913(0.9794-1.0033) | 0.1560 |
|  |  | Simple mode | 4 | 0.9914(0.9697-1.0136) | 0.5014 |
|  |  | Weighted mode | 4 | 0.9920(0.9802-1.0039) | 0.2793 |
|  | Selenium | IVW | 5 | 1.0900(0.9692-1.2257) | 0.1504 |
|  |  | MR Egger | 5 | 1.0847(0.8815-1.3348) | 0.4983 |
|  |  | Weighted median | 5 | 1.1060(0.9773-1.2517) | 0.1104 |
|  |  | Simple mode | 5 | 1.0955(0.8622-1.3919) | 0.4968 |
|  |  | Weighted mode | 5 | 1.1026(0.9693-1.2542) | 0.2116 |
|  | Iron | IVW | 3 | 0.9999(0.9571-1.0446) | 0.9971 |
|  |  | MR Egger | 3 | 0.9996(0.9079-1.1005) | 0.9943 |
|  |  | Weighted median | 3 | 1.0030(0.9598-1.0481) | 0.8939 |
|  |  | Simple mode | 3 | 1.0215(0.9448-1.1044) | 0.6470 |
|  |  | Weighted mode | 3 | 1.0034(0.9587-1.0501) | 0.8986 |
|  | Zinc | IVW | 5 | 1.0241(0.8773-1.1955) | 0.7628 |
|  |  | MR Egger | 5 | 1.2156(1.0390-1.4221) | 0.0927 |
|  |  | Weighted median | 5 | 1.0548(0.9414-1.1818) | 0.3579 |
|  |  | Simple mode | 5 | 0.7180(0.4650-1.1085) | 0.2092 |
|  |  | Weighted mode | 5 | 1.0895(0.9705-1.2232) | 0.2200 |
|  | Copper | IVW | 5 | 1.0741(0.9426-1.2240) | 0.2835 |
|  |  | MR Egger | 5 | 1.2083(1.0288-1.4193) | 0.1045 |
|  |  | Weighted median | 5 | 1.1096(0.9933-1.2396) | 0.0656 |
|  |  | Simple mode | 5 | 0.9398(0.6554-1.3477) | 0.7528 |
|  |  | Weighted mode | 5 | 1.1294(1.0037-1.2708) | 0.1133 |
|  | Vitamin C | IVW | 5 | 0.9673(0.9171-1.0203) | 0.2224 |
|  |  | MR Egger | 5 | 0.9722(0.8842-1.0689) | 0.6005 |
|  |  | Weighted median | 5 | 0.9639(0.9198-1.0102) | 0.1243 |
|  |  | Simple mode | 5 | 0.9675(0.8650-1.0821) | 0.5939 |
|  |  | Weighted mode | 5 | 0.9645(0.9198-1.0114) | 0.2096 |
|  | Vitamin D | IVW | 4 | 1.0058(0.9920-1.0197) | 0.4137 |
|  |  | MR Egger | 4 | 1.0180(1.0015-1.0348) | 0.1652 |
|  |  | Weighted median | 4 | 1.0093(0.9976-1.0211) | 0.1183 |
|  |  | Simple mode | 4 | 1.0035(0.9777-1.0300) | 0.8102 |
|  |  | Weighted mode | 4 | 1.0105(0.9989-1.0224) | 0.1756 |
| IgAN | Calcium | Wald ratio | 1 | 0.9603(0.8934-1.0323) | 0.2720 |
|  | Selenium | IVW | 4 | 0.9894(0.4503-2.1739) | 0.9788 |
|  |  | MR Egger | 4 | 0.4581(0.0005-403.7608) | 0.8424 |
|  |  | Weighted median | 4 | 1.1050(0.5741-2.1268) | 0.7650 |
|  |  | Simple mode | 4 | 1.4551(0.4321-4.8993) | 0.5876 |
|  |  | Weighted mode | 4 | 1.4231(0.5702-3.5518) | 0.5046 |
|  | Iron | IVW | 3 | 1.0655(0.8081-1.4048) | 0.6529 |
|  |  | MR Egger | 3 | 0.7356(0.0382-14.15529) | 0.8722 |
|  |  | Weighted median | 3 | 1.1266(0.8687-1.4612) | 0.3689 |
|  |  | Simple mode | 3 | 1.2170(0.8569-1.7283) | 0.3870 |
|  |  | Weighted mode | 3 | 1.2232(0.8532-1.7537) | 0.3873 |
|  | Zinc | IVW | 4 | 1.1648(0.6944-1.9537) | 0.5632 |
|  |  | MR Egger | 4 | 4.1633(0.0663-261.5059) | 0.5691 |
|  |  | Weighted median | 4 | 1.4154(0.8034-2.4936) | 0.2292 |
|  |  | Simple mode | 4 | 1.4699(0.7554-2.8602) | 0.3392 |
|  |  | Weighted mode | 4 | 1.4628(0.8039-2.6616) | 0.3014 |
|  | Copper | IVW | 4 | 0.8428(0.5260-1.3504) | 0.4771 |
|  |  | MR Egger | 4 | 1.5812(0.0549-45.5683) | 0.8144 |
|  |  | Weighted median | 4 | 0.9236(0.5219-1.6344) | 0.7849 |
|  |  | Simple mode | 4 | 0.9637(0.4858-1.9115) | 0.9223 |
|  |  | Weighted mode | 4 | 0.9522(0.5221-1.7367) | 0.8833 |
|  | Vitamin C | IVW | 4 | 0.9860(0.7940-1.2244) | 0.8986 |
|  |  | MR Egger | 4 | 1.0378(0.1612-6.6813) | 0.9724 |
|  |  | Weighted median | 4 | 1.0357(0.8200-1.3082) | 0.7683 |
|  |  | Simple mode | 4 | 1.1082(0.7906-1.5534) | 0.5931 |
|  |  | Weighted mode | 4 | 1.0855(0.8008-1.4713) | 0.6338 |
|  | Vitamin D | Wald ratio | 1 | 0.9709(0.9041-1.0426) | 0.4164 |
| MN | Calcium | IVW | 5 | 1.0030(0.9956-1.0106) | 0.4266 |
|  |  | MR Egger | 5 | 0.9986(0.9852-1.0121) | 0.8493 |
|  |  | Weighted median | 5 | 1.0001(0.9929-1.0074) | 0.9786 |
|  |  | Simple mode | 5 | 0.9996(0.9873-1.0121) | 0.9563 |
|  |  | Weighted mode | 5 | 0.9997(0.9922-1.0073) | 0.9507 |
|  | Selenium | IVW | 10 | 1.0093(0.9229-1.1038) | 0.8388 |
|  |  | MR Egger | 10 | 1.1243(0.7540-1.6765) | 0.5812 |
|  |  | Weighted median | 10 | 1.0042(0.8943-1.1277) | 0.9431 |
|  |  | Simple mode | 10 | 0.9778(0.8087-1.1824) | 0.8223 |
|  |  | Weighted mode | 10 | 1.0004(0.8445-1.1850) | 0.9963 |
|  | Iron | IVW | 8 | 0.9808(0.9307-1.0337) | 0.4700 |
|  |  | MR Egger | 8 | 1.0268(0.7931-1.3292) | 0.8476 |
|  |  | Weighted median | 8 | 0.9896(0.9449-1.0365) | 0.6593 |
|  |  | Simple mode | 8 | 0.9862(0.9229-1.0538) | 0.6932 |
|  |  | Weighted mode | 8 | 0.9784(0.9174-1.0435) | 0.5273 |
|  | Zinc | IVW | 10 | 1.0137(0.9356-1.0984) | 0.7390 |
|  |  | MR Egger | 10 | 1.0466(0.7326-1.4951) | 0.8088 |
|  |  | Weighted median | 10 | 1.0232(0.9210-1.1367) | 0.6692 |
|  |  | Simple mode | 10 | 1.0102(0.8688-1.1745) | 0.8983 |
|  |  | Weighted mode | 10 | 1.0247(0.8806-1.1925) | 0.7592 |
|  | Copper | IVW | 10 | 1.0091(0.9294-1.0957) | 0.8284 |
|  |  | MR Egger | 10 | 1.1961(0.8271-1.7297) | 0.3693 |
|  |  | Weighted median | 10 | 1.0419(0.9319-1.1648) | 0.4713 |
|  |  | Simple mode | 10 | 1.0499(0.8744-1.2606) | 0.6143 |
|  |  | Weighted mode | 10 | 1.0673(0.91181.2492) | 0.4387 |
|  | Vitamin C | IVW | 10 | 0.9799(0.9454-1.0157) | 0.2674 |
|  |  | MR Egger | 10 | 1.1282(0.9083-1.4014) | 0.3072 |
|  |  | Weighted median | 10 | 0.9739(0.9287-1.0212) | 0.2747 |
|  |  | Simple mode | 10 | 1.0037(0.9394-1.0723) | 0.9159 |
|  |  | Weighted mode | 10 | 0.9631(0.9060-1.0238) | 0.2586 |
|  | Vitamin D | IVW | 5 | 1.0039(0.9979-1.0099) | 0.2025 |
|  |  | MR Egger | 5 | 1.0109(1.0005-1.0213) | 0.1313 |
|  |  | Weighted median | 5 | 1.0065(0.9999-1.0131) | 0.0519 |
|  |  | Simple mode | 5 | 0.9927(0.9797-1.0059) | 0.3387 |
|  |  | Weighted mode | 5 | 1.0069(0.9998-1.0140) | 0.1297 |
| CTIN | Calcium | Wald ratio | 1 | 0.9930(0.9730-1.0135) | 0.5016 |
|  | Selenium | NA | NA | NA | NA |
|  | Iron | NA | NA | NA | NA |
|  | Zinc | NA | NA | NA | NA |
|  | Copper | NA | NA | NA | NA |
|  | Vitamin C | NA | NA | NA | NA |
|  | Vitamin D | Wald ratio | 1 | 1.0168(0.9966-1.0375) | 0.1035 |
| CKD | Calcium | IVW | 5 | 0.9909(0.9761-1.0059) | 0.2324 |
|  |  | MR Egger | 5 | 0.9873(0.9492-1.0270) | 0.5704 |
|  |  | Weighted median | 5 | 0.9952(0.9771-1.0135) | 0.6033 |
|  |  | Simple mode | 5 | 0.9944(0.9719-1.0174) | 0.6535 |
|  |  | Weighted mode | 5 | 0.9962(0.9773-1.0154) | 0.7145 |
|  | Selenium | IVW | 8 | 0.9567(0.7763-1.1789) | 0.6777 |
|  |  | MR Egger | 8 | 2.3208(0.7878-6.8374) | 0.1776 |
|  |  | Weighted median | 8 | 0.9682(0.7490-1.2516) | 0.8052 |
|  |  | Simple mode | 8 | 0.7831(0.5589-1.0973) | 0.1984 |
|  |  | Weighted mode | 8 | 0.9953(0.7288-1.3590) | 0.9770 |
|  | Iron | IVW | 6 | 1.0159(0.9430-1.0944) | 0.6784 |
|  |  | MR Egger | 6 | 0.6335(0.2827-1.4195) | 0.3296 |
|  |  | Weighted median | 6 | 1.0369(0.9413-1.1423) | 0.4624 |
|  |  | Simple mode | 6 | 1.0488(0.9249-1.1893) | 0.4909 |
|  |  | Weighted mode | 6 | 1.0481(0.9359-1.1737) | 0.4530 |
|  | Zinc | IVW | 8 | 0.9222(0.7703-1.1041) | 0.3781 |
|  |  | MR Egger | 8 | 1.2699(0.4836-3.3348) | 0.6449 |
|  |  | Weighted median | 8 | 0.9712(0.7654-1.2324) | 0.8100 |
|  |  | Simple mode | 8 | 1.0151(0.7359-1.4002) | 0.9300 |
|  |  | Weighted mode | 8 | 0.9968(0.7240-1.3726) | 0.9850 |
|  | Copper | IVW | 8 | 1.1111(0.9231-1.3372) | 0.2653 |
|  |  | MR Egger | 8 | 1.2231(0.4509-3.3176) | 0.7061 |
|  |  | Weighted median | 8 | 1.0821(0.8483-1.3804) | 0.5250 |
|  |  | Simple mode | 8 | 1.1143(0.7680-1.6165) | 0.5866 |
|  |  | Weighted mode | 8 | 1.0899(0.7685-1.5456) | 0.6440 |
|  | Vitamin C | IVW | 8 | 1.0388(0.9671-1.1157) | 0.2966 |
|  |  | MR Egger | 8 | 1.4035(0.9964-1.9768) | 0.1005 |
|  |  | Weighted median | 8 | 1.0301(0.9359-1.1336) | 0.5447 |
|  |  | Simple mode | 8 | 1.0153(0.8851-1.1646) | 0.8347 |
|  |  | Weighted mode | 8 | 1.0009(0.8681-1.1540) | 0.9906 |
|  | Vitamin D | IVW | 5 | 0.9883(0.9652-1.0121) | 0.3325 |
|  |  | MR Egger | 5 | 1.0154(0.9551-1.0795) | 0.6581 |
|  |  | Weighted median | 5 | 0.9937(0.9732-1.0147) | 0.5562 |
|  |  | Simple mode | 5 | 0.9888(0.9595-1.0191) | 0.5053 |
|  |  | Weighted mode | 5 | 0.9977(0.9769-1.0190) | 0.8432 |
| Cystic kidney disease | Calcium | Wald ratio | 1 | 1.0242(0.9984-1.0381) | 0.1048 |
|  | Selenium | Wald ratio | 1 | 1.0134(0.9377-1.0953) | 0.7362 |
|  | Iron | NA | NA | NA | NA |
|  | Zinc | Wald ratio | 1 | 0.9851(0.9190-1.0560) | 0.6719 |
|  | Copper | Wald ratio | 1 | 0.9684(0.9019-1.0398) | 0.3761 |
|  | Vitamin C | Wald ratio | 1 | 0.9999(0.9742-1.0264) | 0.9975 |
|  | Vitamin D | Wald ratio | 1 | 0.9963(0.9832-1.0096) | 0.5868 |

NA indicates that there are no eligible SNPs after analyzing through the set threshold. Abbreviation: HTN, Hypertensive Nephropathy; DKD, Diabetic Kidney Disease; IgAN, IgA Nephropathy; MN, Membranous Nephropathy; CTIN, Chronic Tubulointerstitial Nephritis; CKD, Chronic Kidney Disease; IVW, Inverse Variance Weighted.
